# Burkitt lymphoma: epidemiological features and survival in a South African centre

**DOI:** 10.1186/1750-9378-9-19

**Published:** 2014-06-10

**Authors:** Daniela C Stefan, Rabeen Lutchman

**Affiliations:** 1 Department of Paediatrics and Child Health, Tygerberg Hospital and Stellenbosch University, Tygerberg, PO Box 19063, Cape Town, South Africa

**Keywords:** Burkitt lymphoma, Sporadic, Endemic, Epidemiological features, Survival, South Africa

## Abstract

**Background:**

The epidemiology of Burkitt Lymphoma (BL) shows that the endemic type is mainly confined to equatorial Africa and has a very close association with the Epstein-Barr virus (EBV), while the sporadic variant shows only a 20% association with EBV and is seen mainly in Europe and North America. An immunodeficent form of BL has been described more recently. This study aimed to describe the epidemiological characteristics and survival of children presenting with BL to Tygerberg Hospital, Cape Town, in South Africa.

**Methods:**

A retrospective, descriptive study reviewed all pediatric cases of Burkitt lymphoma at Tygerberg Hospital Oncology Unit between 1 January 1995 and 31 December 2010. The following data were analysed: age at diagnosis, gender, anatomic site, race, socio-economic demographic (rural vs. urban), treatment protocol, side effects, viral characteristics and survival. All cases were confirmed by histology and reviewed by a tumour board.

**Results:**

A total of 51 patients with Burkitt lymphoma were analysed from 1995 to 2010. Their age ranged from 2 to 14 years (mean of 6.8 years).The male to female ratio was 3.6/1. Most of the patients lived in an urban setting (52.9%). The initial presenting tumour site was abdominal in most cases (76.4%). The majority of patients (90%) were treated with the LMB protocol. Neutropenic sepsis, mucositis and gastroenteritis were the top 3 side effects while receiving therapy (58.8%, 50.9% and 31.3% respectively). The overall survival rate was 64.7%. A documented positive HIV1 test was found in 11% of the total number of patients. The stage of the disease at the time of presentation strongly influenced the outcome with only 41.6% of stage 4 patients surviving (p = 0.03).

**Conclusions:**

The patients seen at Tygerberg Hospital, South Africa presented typically with the sporadic variant of Burkitt Lymphoma. The patients presented with large abdominal masses and in an advanced stage of the disease.

## Introduction

Burkitt Lymphoma (BL) is common amongst children, especially in Africa. BL accounts for 30 to 50% of all childhood cancer in equatorial Africa with an estimated incidence of 3 to 6 cases per 100,000 children per year [1]. The incidence of BL in Africa is approximately 50-fold higher than that seen in the United States (US) [2].It was first described in Eastern Africa as a sarcoma of the jaw [3].

The three clinical variants of BL are classified as endemic, sporadic and immunodeficient, according to their epidemiological characteristics.

The peculiar epidemiology of BL showed that the endemic type was mainly confined to equatorial Africa and has a very close association with the Epstein–Barr virus (EBV), with approximately 95% of the ‘endemic’ cases showing the presence of the EBV genome in their tumour cells. The endemic form typically presents in younger children with jaw masses and often spreads to the central nervous system.

BL is not restricted to Eastern Africa, it occurs elsewhere too, but with a lesser incidence. This was classified as the sporadic form of the tumour. The tumours found outside the Central African Belt (according to the WHO, the BL belt extends from East to West between 10 degrees north and 10 degrees south of the equator, continuing along the eastern coast of Africa) showed also different viral characteristics – EBV being present only in 20% of cases [4].

Research has shown human immunodeficiency virus (HIV-1) to be significantly associated with BL [5]. Such cases are classified as immunodeficiency BL and can also be associated with organ transplantation, secondary to immunosuppressive drugs. BL can, in fact, be one of the diseases associated with the initial manifestation of AIDS [6]. Almost half of the patients (40 – 50%) of HIV–associated BL are positive for EBV [6].

In South Africa, the immunodeficient form of BL is seen with increasing frequency. The literature is controversial with regard to the predominance of the other two forms, endemic and sporadic BL, in children: older studies published found that patients closely resembled endemic BL while more recent literature indicates that children present the sporadic variant [6-8].

This study aimed to describe the demographic characteristics of the patients diagnosed and treated for BL in the pediatric oncology unit at Tygerberg Hospital, Cape Town, South Africa and to examine the similarities and differences between sporadic Burkitt lymphoma and endemic Burkitt lymphoma. In addition to the epidemiological features, the treatment protocol used, the adverse effects, relapse, association with HIV infection and correlation of the stage with ethnic groups and survival were analysed.

## Methodology

The study population included all paediatric patients (<15 years) diagnosed with BL (confirmed by histology or cytology by a pathologist) and managed in the pediatric oncology unit, from 1 January 1995 to 31 December 2010.

Data extracted from the records included the patient’s birth date, age at diagnosis, sex, ethnicity, anatomical site of the primary tumour, tumour stage, systems involved by tumour and site of metastases and HIV-1 serology results.

Data were taken out from the local tumour registry of the unit and the findings were correlated with the national childhood cancer registry. All cases were confirmed by biopsy and histopathology.The following markers were used: CD45, CD3, CD20, bcl-6, CD10, TdT & ki-67. Molecular tools were not used as they became available only in the last few years.

The treatment protocol used was based on the French Paediatric Oncology Society protocol LMB – 89 [9]. All patients were treated for prevention or diagnosis of tumour lysis syndrome with allopurinol, hyperhydration and urinary alkalisation at the start of the induction.

Staging investigations done to assess the extent of disease, included imaging studies (chest radiography, abdominal ultrasound), lumbar puncture with CSF evaluation as well as bilateral bone marrow aspirate and trephine. Some of the children had computerized tomography scans. The stage was determined according to the St Jude Staging system.

Adverse effects of the treatment were recorded.

The 5 year overall survival was calculated from the diagnosis date. All patients were followed up at the same clinic.

From 2001 onwards all children were tested for HIV infection as a routine examination. Data were missing regarding results prior to this date.

The extracted data was recorded in Excel format. Descriptive statistics including frequency tables, histograms, means and standard deviations were performed. Data were compared using the chi square test, ANOVA or cross tabulations depending on the type of data.

### Ethical approval

Ethical approval for this study was obtained from the Health Research Ethics Committee of the University of Stellenbosch; ethics reference number N11/03/100. The manager of Tygerberg Hospital approved the retrieval of data from the Tygerberg Hospital folders.

## Results

A total of 51 patients with histologically confirmed Burkitt Lymphoma were reviewed from 1 January 1995 to 31 December 2010.

### Age and ethnicity

The age group of the patients ranged from 2 to 14 years with a median age of 6 years and a mean of 6.8 years (SD = 3.82). Coloured or mixed ancestry patients were the majority of the patients, 62.7% (n = 32), white patients represented 19.6% (n = 10), and black patients, 17.6% (n = 9). The “Cape coloured” are the largest population group in Western Cape and they are historical descendants of couples of distinct ethnicity. The age at diagnosis for the coloured patients ranged from 2 to 14 years with a mean age of 6.6 years; it was 3 to 14 years for the white population and 3 to12 years with a mean 5.6 years for the black children. The mean age of the different ethnic groups was not statistically significant (p > 0.1).

#### Gender

There were 40 males and 11 females with an M : F ratio of 3.6:1 equally represented in all ethnic groups.

#### Social demographic

Of the all patients, 52.9% lived in an urban setting while 47.1% came from a rural environment. Urban setting was defined as living in a city and rural as living in a village or sharing the characteristic of farming or country life. The rural setting was also correlated with an average income of less than 400$ per family per month.

#### Tumour

**Figure 1:**
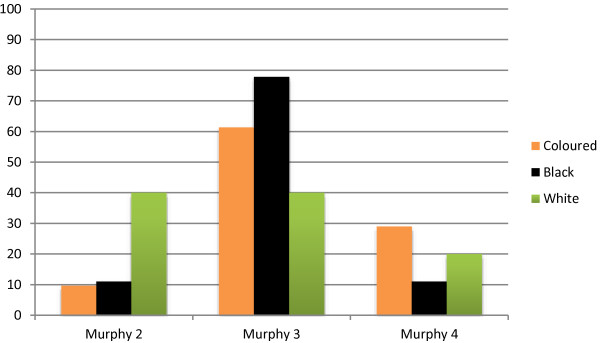
Stage of tumour correlated with ethnic group.

There was a predominance of abdominal primary tumours (76.4%). A further 3.9% presented with a pelvic mass. Only 11.7% presented with a head/neck mass showing a difference from endemic Burkitt tumours found on the African Belt. All ethnic groups presented predominantly with abdominal tumours and there was no statistical significance between the distributions of various sites of the primary tumours in the groups (p > 0.1).The majority of patients presented late with the disease with 58% in Murphy Stage 3 and 23% in Murphy Stage 4. Lung and bone marrow metastases were most frequent (29.4% and 25.4% respectively) while 88% of the patients had abdominal involvement either as a primary site or with metastases. When comparing ethnicity to the clinical stage in which patients presented, 90% of the coloured population and 88% of the black population presented at a more advanced stage (i.e. 3 or 4) compared to the white population, where 60% presented in an advanced stage (p =0.06) (Figure 1).

#### Treatment and survival

**Figure 2:**
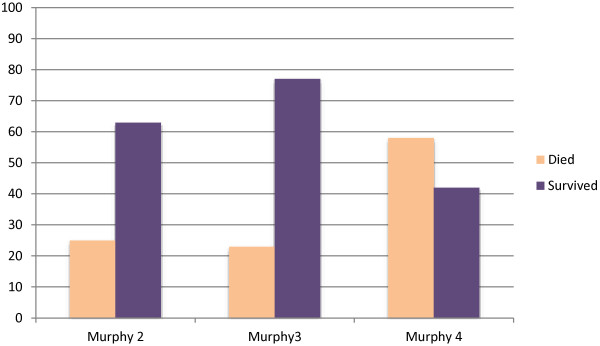
Stage of tumor versus survival.

The overall five year survival rate was 64.7%. The majority of patients (90%) were treated with the LMB protocol 1989 and 1996. Only 1 patient died before treatment was started and 2 received only Vincristine and Prednisone before demise. A total of 4 patients relapsed (3 in stage 3 of disease and 1 in stage 4). The sites of relapses were in the bone marrow (3 cases) and in the lung (1 case).The stage of the disease at the time of presentation strongly influenced the outcome with only 41.6% of stage 4 patients surviving, versus 62% in stage II and 78% in stage III (p = 0.03) (Figure 2). Neutropenic sepsis, mucositis and gastroenteritis were the top 3 side effects patients suffered while receiving therapy (58.8%, 50.9% and 31.3% respectively).

Gender did not have an influence on survival (p > 0.1).

Race did not have an influence of side effect profile or the relapse rate and for patients who did present with side effects, survival was not significantly affected (both p > 0.1).

Race influenced the outcome of the disease as black children presented with more advanced disease than white children and the survival between the groups differed markedly.

#### Viral characteristics

Six out of the 15 patients tested for HIV had a documented positive result. Only 50% of these patients survived. The patients with HIV presented with severe illness, with two out of the six demising before the start of the full chemotherapy protocol or antiretroviral. They were all classified as WHO stage 4 HIV disease. The CD4 count ranged from 304 to 1194 with a mean of 612 and median of 394. Two patients were on antiretrovirals at the time of diagnosis and two were started after the diagnosis was made. All of them had complications.

## Discussion

The findings from this study show that children with BL seen at Tygerberg Hospital (Western Cape, South Africa) present with clinical features that are more in keeping with sporadic BL. This differs to what was previously described by *Hesseling et al.* in 1989 which found that the characteristics of the 22 patients in their study were similar to African endemic BL [8].In that study 13 of the 22 cases (59%) showed involvement of the jaws which is in keeping with what is described as endemic BL.

As South Africa is a diverse country with multiple population groups with different ethnic and cultural background, the demographics of the patients that were included in this study reflect largely the greater population of the Western Cape (predominantly a coloured population – 53.9% followed by the black and white population – 26.7 and 18.4 respectively) [10]. This is different and unique to the rest of South Africa which has a predominant black population – 79% [10]. Our study analyzed possible differences related to BL and ethnicity and found that all ethnic groups presented predominantly with abdominal tumours. There was no statistical significance in the distribution of sites of the primary tumours within the various ethnic groups (p > 0.1).

Of note, coloured and black children presented with more advanced disease than the white children and the survival ratios between the groups differ markedly.

Coloured and black children with BL presented at a much younger age (5–6 years) compared to the white population at 8–9 years.

The younger age presentation is well documented in endemic BL, while the older child /younger adolescent presentation is seen more in sporadic BL [11,12]. This study revealed an age difference between ethnic groups, suggesting that the white population has a tendency to follow the sporadic pattern.

This cohort displays similar trends in gender distribution as seen previously.

Male predominance was reported in both types of the epidemiological variants of BL. Several studies from Israel, Netherlands, Pakistan and Yemen [13-16] showed that gender did not have an influence on the prognosis of survival. Even though males had higher tumour risk both males and females had similar outcome.

Denis Burkitt first described this tumour in 1958 and later published epidemiological investigations revealing that it was found in humid, hot, low lying areas, referring to the geographic distribution as the “tumour safari”. The tumour was confined to the African Belt, which extends from east to west between 10 degrees north and 10 degrees south of the equator, continuing along the eastern coast of Africa. The patients in this study all came from the Tygerberg Hospital’s drainage area (this hospital serves the immediate surrounding areas, providing primary and secondary health care to children, as well as tertiary care to all paediatric patients in the eastern parts of Cape Town and the Northern and Eastern rural districts of the Western Cape, as well as to selected cases from Namibia). The patient demographic for this study showed a negligible difference between the numbers of patients from rural and urban background (52.9% and 47.1% respectively). Many patients reside in very dry, and relatively colder climates where the temperature drops below 15°C (60°F), showing a marked difference from the endemic BL climate pattern, where the average temperature ranges between 18-27°C with a humidity of 30% or higher. The socioeconomic status had no influence on the incidence of the tumour.

All ethnic groups predominantly presented with abdominal involvement. Jaw (head and neck) tumours are a characteristic feature of BL on the African Belt and are found in approximately 50% of the cases. These tumours have also been seen to be age dependent and generally are found in the younger African child. In our study, ethnicity had no significant effect on the site of the primary tumour.

Numerous studies have shown the repeated association between EBV and the *c-myc* oncogene in the pathogenesis of eBL [6,10]. On-going studies and current literature show a link between *Plasmodium falciparum* and EBV reactivation as recognised co-factors in the genesis of endemic BL [12,13]. There is clear evidence regarding the role of EBV and malaria in the pathogenesis of endemic BL and on its association with HIV-1, but insufficient data to support the role of such factors in sporadic cases, where EBV is seen in only about 20% of the patients [4].

This area is also not endemic for malaria and there were no documented malaria cases showing that in this region *Plasmodium falciparum* did not play a role in BL pathogenesis in our study population.

It was clear that when this tumour was complicated by HIV1 the disease presentation was more severe and the progression and the outcome were worsened. Of the two patients who were on ARVs at the time of diagnosis, one survived and the other demised and a similar outcome was seen in the two patients who were started on ARVs during the illness.

A previous study done in 2011 in South Africa, analysed the clinical features, the management and prognosis of 2 cohorts of BL: HIV infected children compared to HIV non-infected children That study showed that, despite a similar distribution by age, sex and stage of disease between the 2 groups, the mortality ratio was significantly higher in the HIV positive group [17].

Further investigations and studies are definitely warranted to investigate the effect of ARVs.

The movement away from aggressive surgery and less intensive chemotherapy towards more intensive chemotherapy and a more conservative surgical approach has been shown to be a better management strategy with improved survival outcomes [18] This forms the core of the LMB protocol that was used in the treatment of the majority of patients in this study. The more intensive chemotherapy results in a greater morbidity of the patients with the main concern being neutropenic sepsis. The high rate of neutropenic sepsis seen on this protocol stresses the need for strict isolation facilities and barrier precautions, which are often absent or inadequate in resource poor settings. The complications of the protocol were seen in all stages of the tumour. Ethnicity also had no influence on the side effect profile, with previously disadvantaged ethnic groups with poorer social circumstances having similar morbidity when compared to ethnic groups with better social circumstance.

The overall survival rate of the patients in this study at 64.7% was comparatively lower than others countries who have sporadic BL where more than 80% survival rates are achieved. The major impact on survival came from the stage of the disease at the time of presentation. In first world countries cancer-surveillance programs are more prominent and patients tend to seek medical attention much earlier. The management of the morbidity of the chemotherapy complications also contributes to the vast difference in the overall survival rate.

This study illustrates the importance of early cancer diagnosis of BL in South Africa and the need for increase awareness and education. Late and advanced presentation of cancers impacts on treatment, prognosis and ultimately survival. Just over 80% of patients presented with Murphy Stage 3 and 4 combined. The previously disadvantaged ethnic groups of South Africa suffered the brunt of the impact illustrating the lack of education and delay in seeking early medical advice.

### Limitations of the study

The overall limitations of this study include the analysis of a retrospective data, a small number of patients, missing information on viral studies and multiple statistical comparisons.

## Conclusion

The clinical presentation of the patients seen at Tygerberg Hospital, South Africa from 1995 till 2010 was rather suggestive of sporadic variant of Burkitt Lymphoma with abdominal tumours most frequently seen.

Advanced disease at presentation associated with HIV infection contributed to a poorer outcome of the patients. No difference in outcome was noted between urban and rural settings. Survival rates remain low compared to the reported figures from developed countries mainly due to advanced disease at presentation.

The LMB treatment protocol has a good outcome but is complicated by significant morbidity. Oncology units must be equipped with isolation facilities and intensive infection control procedures must be in place to deal with the high morbidity seen during the management of these tumours.

Strong recommendations are warranted for early recognition of childhood cancers as this has a major impact on outcome and survival. Education programmes to recognise the early signs of cancer are essential and must take place on all levels from the uneducated rural public to the medical students and general practitioners.

## Competing interests

Both authors declare that they have no competing interests.

## Authors’ contributions

DCS- study conception and design, analysis and interpretation of data, drafting of manuscript and critical revision. RL- acquisition of data, analysis and interpretation of data, drafting of manuscript and critical revision. Both authors read and approved the final manuscript.

## References

[B1] Magrath I Epidemiology: clues to the pathogenesis of Burkitt lymphoma Br J Haematol 2012 156 744 10.1111/j.1365-2141.2011.09013.x 22260300

[B2] Ogwang MD Bhatia K Biggar RJ Mbulaiteye SM Incidence and geographic distribution of endemic Burkitt lymphoma in northern Uganda revisited Int J Cancer 2008 123 2658 10.1002/ijc.23800 18767045 PMC2574984

[B3] Burkitt D A sarcoma involving the jaws in African children Br J Surg 1958 46 218 223 10.1002/bjs.18004619704 13628987

[B4] Burkitt D Determining the climatic limitations of a children’s cancer common in Africa Br Med J 1962 2 1019 1022 10.1136/bmj.2.5311.1019 14017064 PMC1926328

[B5] Bornkamm GW Epstein-Barr virus and the pathogenesis of Burkitt’s lymphoma: more questions than answers Int J Cancer 2009 124 8 1745 1755 10.1002/ijc.24223 19165855

[B6] Omar FE Childhood Lymphomas – a brief overview CME 2010 27 7 332 336

[B7] Behrman RE Kliegman RM Jenson HB Nelson Textbook of Pediatrics 2005 17 Philadelphia: Elsevier Saunders

[B8] Hesseling P Wood RE Nortje CJ Mouton S African Burkitt’s lymphoma in the Cape province of South Africa and in Namibia Oral Surg Oral med Oral Pathol 1989 68 2 162 166 10.1016/0030-4220(89)90186-2 2789358

[B9] Patte C Auperin A Michon J Behrendt H Leverger G Frappaz D Lutz P Coze C Perel Y Raphaël M Terrier-Lacombe MJ The Societe Francaise d’Oncologie Pediatrique LMB-89 protocol: highly effective multi-agent chemotherapy tailored to the tumour burden and initial response in 561 unselected children with B-cell lymphoma and L3 leukaemia Blood 2001 97 3370 3379 10.1182/blood.V97.11.3370 11369626

[B10] Statistical release P 0302 (Mid- year population estimates 2013) http://www.statssa.gov.za (accessed last 31 December 2013)

[B11] Emmanuel B Kawira E Ogwang MD Wabinga H Magatti J Nkrumah F Neequaye J Bhatia K Brubaker G Biggar RJ Mbulaiteye SM Age-specific risk and correlations with malaria biomarkers Am J Trop Med Hyg 2011 84 3 397 401 10.4269/ajtmh.2011.10-0450 21363976 PMC3042814

[B12] Donati D Guerreiro-Cacais AO Levitsky V Chen Q Falk KI Orem J Kironde F Wahlgren M Bejarano MT A molecular link between malaria and Epstein-Barr virus reactivation PLoS Pathog 2007 3 6 e80 10.1371/journal.ppat.0030080 17559303 PMC1891325

[B13] Eldar AH Futerman B Abrahami G Attias D Barak AB Burstein Y Dvir R Gabriel H Horovitz J Kapelushnik J Kaplinsky H Miskin H Sthoeger D Toren A Vilk-Revel S Weintraub M Yaniv I Linn S Arush MB Burkitt lymphoma in children: the Israeli Experience J Pediatr Hematol Oncol 2009 31 6 428 436 10.1097/MPH.0b013e31819a5d58 19648792

[B14] Boerma EG van Imhoff GW Appel IM Veeger NJ Kluin PM Kluin-Nelemans JC Gender and age- related differences in Burkitt lymphoma- epidemiological and clinical data from The Netherlands.*Eu J* Cancer 2004 40 18 2781 2787 10.1016/j.ejca.2004.09.004 15571961

[B15] Fadoo Z Belgaumi A Alam M Azam I Naqvi A Pediatric Lymphoma: A 10 year experience at a tertiary care hospital in Pakistan J pediatr Hematol Oncol 2010 32 1 e14 e18 10.1097/MPH.0b013e3181bdf1f3 20051771

[B16] Al-Samawi AS Aulaqi SM Al-Thobhani AK Childhood Lymphomas in Yeman. Clinicopathological Study Saudi Med J 2009 30 9 1192 1196 19750266

[B17] Stefan DC Stones D Newton R Burkitt lymphoma in South African children: One or two entities? Transfus Apher Sci 2011 44 191 194 10.1016/j.transci.2011.01.020 21345736

[B18] Davidson A Desai F Hendricks M Hartley P Millar A Numanoglu A Rode H The evolving management of Burkitt’s lymphoma at Red Cross Children’s Hospital S Afr Med J 2006 96 9 Pt 2 950 954 17077923

